# Degradation of Hydrocarbons and Heavy Metal Reduction by Marine Bacteria in Highly Contaminated Sediments

**DOI:** 10.3390/microorganisms8091402

**Published:** 2020-09-11

**Authors:** Filippo Dell’Anno, Christophe Brunet, Leonardo Joaquim van Zyl, Marla Trindade, Peter N. Golyshin, Antonio Dell’Anno, Adrianna Ianora, Clementina Sansone

**Affiliations:** 1 Stazione Zoologica Anton Dohrn, Istituto Nazionale di Biologia, Ecologia e Biotecnologie Marine, Villa Comunale, 80121 Napoli, Italy; filippo.dellanno@szn.it (F.D.); christophe.brunet@szn.it (C.B.); adrianna.ianora@szn.it (A.I.); 2 Department of Biotechnology, Institute for Microbial Biotechnology and Metagenomics (IMBM), University of the Western Cape, Bellville 7535, Cape Town, South Africa; lvanzyl@uwc.ac.za (L.J.v.Z.); ituffin@uwc.ac.za (M.T.); 3 Centre for Environmental Biotechnology (CEB), School of Natural Sciences, Bangor University, Gwynedd LL57 2UW, UK; p.golyshin@bangor.ac.uk; 4 Dipartimento di Scienze della Vita e dell’Ambiente, Università Politecnica delle Marche, Via Brecce Bianche, 60131 Ancona, Italy; a.dellanno@univpm.com

**Keywords:** bioremediation, PAHs, heavy metals, bacteria, pollution, sediments

## Abstract

Investigations on the ability of bacteria to enhance removal of hydrocarbons and reduce heavy metal toxicity in sediments are necessary to design more effective bioremediation strategies. In this study, five bacterial strains, *Halomonas* sp. SZN1, *Alcanivorax* sp. SZN2, *Pseudoalteromonas* sp. SZN3, *Epibacterium* sp. SZN4, and *Virgibacillus* sp. SZN7, were isolated from polluted sediments from an abandoned industrial site in the Gulf of Naples, Mediterranean Sea, and tested for their bioremediation efficiency on sediment samples collected from the same site. These bacteria were added as consortia or as individual cultures into polluted sediments to assess biodegradation efficiency of polycyclic aromatic hydrocarbons and heavy metal immobilisation capacity. Our results indicate that these bacteria were able to remove polycyclic aromatic hydrocarbons, with a removal rate up to ca. 80% for dibenzo-anthracene. In addition, these bacteria reduced arsenic, lead, and cadmium mobility by promoting their partitioning into less mobile and bioavailable fractions. Microbial consortia generally showed higher performance toward pollutants as compared with pure isolates, suggesting potential synergistic interactions able to enhance bioremediation capacity. Overall, our findings suggest that highly polluted sediments select for bacteria efficient at reducing the toxicity of hazardous compounds, paving the way for scaled-up bioremediation trials.

## 1. Introduction

Pollutants, such as heavy metals and metalloids, polycyclic aromatic hydrocarbons (PAHs), and halogenated compounds are frequently released into the environment through improper industrial discharges or waste disposal practices, incomplete combustion of organic matter [1] and continental runoff [2]. Compounds released from human activities pose severe threats to ecosystem health [3], notably coastal or transitional ecosystems, as well as systems with low hydrodynamics [4]. PAHs and heavy metals, such as arsenic [5], cadmium [6], chromium [7], lead [8], and mercury [9], have been reported to affect biological systems, such as cell membranes or organelles to enzymes involved in metabolism, detoxification, and DNA damage repair [10]; thus, causing cell cycle modulation, carcinogenesis, or apoptosis [11,12]. Accumulation of pollutants also affects microbial taxa composition and, thus, biodiversity and ecosystem functioning, with potential cascade effects on the provisioning of ecosystem goods and services for human wellbeing [13].

Polluted areas represent a threat for the environment and restoration of these habitats is therefore a challenge for humanity and science. Various solutions based on chemical and electrochemical strategies have been developed for the remediation of contaminated marine sediments, such as reverse osmosis, electro dialysis, ultrafiltration, ion exchange, and chemical precipitation [14]. Unfortunately, these methods have several disadvantages such as high costs, the generation of toxic sludge [15] and the inability to apply many of these techniques in situ. For such reasons, international policies (e.g., the European Marine Strategy Framework Directive) are increasingly seeking alternative solutions limiting sediment handling interventions and promoting the decontamination of these matrices by using eco-compatible in situ technologies.

Bioremediation strategies employing microorganisms for the remediation of contaminated environmental matrices [16] are a promising alternative. Indeed, their efficiency in reducing contamination levels is noteworthy, together with their versatility to be used with different types of contaminants and in different environmental contexts [17].

Bioremediation mechanisms occur under aerobic or anaerobic conditions. The degradation of organic pollutants involves aerobic/anaerobic respiration and fermentative metabolism while transformation/sequestration of heavy metals (which do not undergo degradation) are based on bioaccumulation, biotransformation, and bioleaching activities [18]. Generally, bioremediation processes can be enhanced by biostimulation of autochthonous assemblages (e.g., by adding different chemical compounds and or electron donors/acceptors) or by bioaugmentation, which consists of adding selected microorganisms capable of degrading or mobilizing contaminants [19].

A bioaugmentation approach is challenging to identify efficient microorganisms with sufficient bioremediation capability and to investigate their performance for the decontamination of polluted marine sediments.

In the present study, we isolated and identified different bacterial strains from sediment samples collected in front of an abandoned industrial site located in the Gulf of Naples (Mediterranean Sea), characterised by concentrations of Cu, Fe, Hg, Mn, Ni, Pb, Zn, as well as polychlorobiphenyls (PCBs), polycyclic aromatic hydrocarbons (PAHs), and dichlorodiphenyltrichloroethane (DDT) that are above the legal limits [20,21], and tested the isolated taxa for their bioremediation potential on sediment samples in terms of hydrocarbon degradation and decrease in metal mobility.

The research hypothesis of the study assumes that such bacterial strains are adapted to harsh environmental conditions, and therefore may be effective for bioremediation of polluted sediments based on a bio augmentation approach. We also aimed to compare their bioremediation potential as single isolates or as a mixture (consortia) of species. Our study focuses on both hydrocarbons and heavy metals, determining the removal capacity of nine chemical species of hydrocarbons as well as the mobility of metals (five different elements) following treatment of contaminated sediments with bacteria. This strategy will allow to select the most efficient consortia/bacteria affecting both hydrocarbons and metal pollution in sediments, providing new insights for a better understanding of the biotechnological potential of natural consortia or single bacteria that could be transferred to further scaled up ex situ bioremediation.

## 2. Materials and Methods

### 2.1. Sediment Sampling

**Figure 1:**
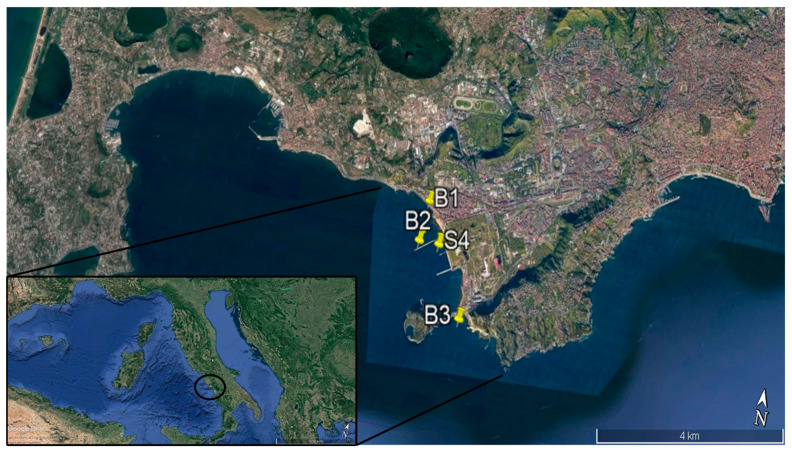
The sampling sites in the Gulf of Naples (Mediterranean Sea). B1, B2, B3 correspond to the stations used for sampling sediments for isolation of bacteria. S4 corresponds to the station where sediments were sampled for microcosm experiments.

The sediment samples used for the isolation of bacteria were collected in November 2017 with a Van Veen grab from three stations located in the Bagnoli–Coroglio area (Gulf of Naples): 40.81555 N, 14.16075 E (B1); 40.80834 N, 14.15966 E (B2); and 40.79644 N, 14.17293 E (B3) (Figure 1). Duplicate samples were immediately placed into sterile bags (Whirl-Pak, Nasco, Fort Atkinson, WI, USA) and stored at 4 °C in the dark, until their processing in the laboratory. Additional sediment samples were collected at one of the three stations (i.e., station S4, Figure 1) and used for bioremediation experiments using a bioaugmentation approach (i.e., adding the previously isolated and identified bacterial strains).

### 2.2. Bacterial Strain Isolation

To select the most promising bacterial strains for bioremediation purposes, bacterial colonies were isolated by plating 500 µL (dilution 1:10) of the sediment onto marine agar (MA) (Difco Laboratories Incorporated, Franklin Lakes, NJ, USA) in the presence of three different metals: Pb^2+^ (500 µg mL^−1^), As^3+^ (500 µg mL^−1^), Cd^2+^ (10 µg mL^−1^), and incubated at 28 °C for 7 days.

Marine agar, a complex growth medium used for the cultivation of heterotrophic marine bacteria, was chosen to select cultivable bacteria in order to evaluate their potential biotechnological use for bioremediation purposes.

Metals and metalloids were selected since As and Cd [22,23] are among the most toxic compounds and, along with Pb, are highly distributed in the studied area [20]. The colonies observed on the agar after 48 h occurred naturally as mixed cultures. The consortia appear on the marine agar plates mainly formed by five different colonies.

- The first appears as white/yellow with a smooth circular raised form.
- The second appears with a white colour with smooth flat margins.
- The third appears with a white colour, with rough, circular, irregular flat margins.
- The fourth appears with a light yellow colour, with a circular convex form and entire margins.
- The fifth appears with a cream colour, with a rough and elevated form, with regular margins.

To obtain pure cultures, the colonies were re-plated on marine agar through serial dilution to obtain single colonies. After 48 h of incubation, the different colony morphologies were observed and re-streaked until confirmed pure. Once isolated, colonies were suspended in 30% sterile glycerol and stored at −80 °C.

### 2.3. Identification of Bacterial Isolates

To identify the bacterial isolates, PCR analysis was conducted using the universal bacterial primers E9F (5′-GAGTTTGATCCTGGCTCAG-3′) and U1510R (5′-GGTTACCTTGTTACG- ACTT-3′; [24]) targeting 16S rRNA genes. All polymerase chain reactions (PCR) were carried out in a Perkin Elmer Thermocycler (Gene Amp PCR system 6700) in a 50 µL reaction volume containing 1× PCR buffer, 200 µM of each dNTP, 0.5 µM of each primer, 0.2 U of Taq Gold polymerase (Applied Biosystems, Waltham, MA, USA) and 1 µL of template DNA. Thermal cycling conditions were 5 min denaturation at 94 °C; 30 cycles of 94 °C for 30 s, 55 °C for 30 s and 72 °C for 90 s; final elongation step at 72 °C for 5 min. Each amplification mixture was analysed by agarose gel (1.2% *w/v*) electrophoresis in TAE (TRIS-acetate-EDTA) buffer solution (0.04 M Tris-acetate, 1 mM Ethylenediaminetetraacetic acid, EDTA) containing 0.5 μg mL^−1^ (*w/v*) ethidium bromide. The PCR products were purified and sequenced at the Central Analytical Facility at Stellenbosch University (Stellenbosch, South Africa) using an ABI PRISM 377 automated sequencer (Applied Biosystems). The data from the sequencing unit was processed using Chromas Pro v. 1.5a software (Technelysium, South Brisbane, QLD, Australia) for alignment and manual editing of sequences. The consensus sequences of the isolates were compared with those deposited in GenBank using the BLAST program. The neighbour-joining phylogenetic tree was constructed using MEGA v. 7 software [25]. Bootstrap tests were performed with 1000 pseudo replicates.

### 2.4. Experimental Setup

The experimental setup consisted of 27 flasks (12 for the four consortia and 15 for the five single isolates), plus three flasks as control containing only sediments and the culture medium Marine Broth (MB, Difco Laboratories Incorporated, Franklin Lakes, NJ, USA). The treatment flasks were filled with sediment, MB and bacteria as follows: 50 mL of MB containing bacteria at a concentration of about 2 × 10^6^ cells mL^−1^ were incubated in TPP tissue culture flasks (500 mL), together with 50 g of contaminated sediments.

The flasks were mixed manually every 12 h. The sediment colour was almost black due to oil spilled during the years and the Eh measurements confirmed its oxic state. The other three flasks were used as controls and filled with sediment and culture medium Marine Broth at the same concentrations as the experimental flasks. All experiments were carried out in triplicate.

Flasks were incubated for 27 days at 28 °C in the dark and 20 mL samples (sediment and broth, ratio 1:1) were taken immediately after incubation (time 0) and at the end of the incubation period (i.e., after 27 days) for the analysis of hydrocarbon concentrations and the determination of heavy metal content and their partitioning in the different geochemical fractions of the sediments. The incubation time was selected on the basis of the growth curve of the bacteria [26].

### 2.5. Hydrocarbons and Heavy Metal Analyses

Determination was carried out by gas chromatography–mass spectrometry (GC-MS), according to the 8270 Environmental Protection Agency (EPA) method and 6020 EPA method described in [27,28]. More specifically, following the method described in [28], samples were prepared for analysis by GC-MS using the appropriate sample preparation and sample clean up procedures. The semivolatile compounds were introduced into the GC/MS by injecting the sample extract into a GC equipped with a narrow-bore fused-silica capillary column. The GC column is temperature-programmed to separate the analytes, which were then detected with an MS connected to the GC. Analytes eluted from the capillary column that were introduced into the MS via a direct connection. Identification of target analytes was accomplished by comparing their mass spectra and retention times (RT) with the mass spectra and RTs of known standards for the target compounds. Quantitation was accomplished by comparing the response of a major (quantitation) ion relative to an internal standard (IS) using an appropriate calibration curve for the intended application.

The method in [27] describes multi-element determinations using Inductively coupled plasma mass spectrometry (ICP-MS) in environmental samples. The method measures ions produced by a radio frequency inductively coupled plasma. Analyte species in liquid were nebulised and the resulting aerosol was transported by argon gas into the plasma torch. The ions produced by high temperatures were entrained in the plasma gas and introduced, by means of an interface, into a mass spectrometer. The ions produced in the plasma were sorted according to their mass-to-charge (m/z) ratios and quantified with a channel electron multiplier. Interferences were assessed and valid corrections applied. Interference correction included compensation for background ions contributed by the plasma gas, reagents, and constituents of the sample matrix.

Finally, metal distributions in different mineralogical fractions were determined by means of a selective extraction procedure, which utilised, sequentially, specific chemical reagents to extract heavy metals associated with different geo-chemical phases (i.e., carbonate/exchangeable, oxidisable, reducible, and residual fraction; [29]).

### 2.6. Statistical Analysis

Statistical significance of the experimental results (mean and SD of the triplicate) was tested using Student’s t-test (mean comparison) and Fisher–Snedecor test (variance comparison), using PAST3 software (http://folk.uio.no/ohammer/past; accessed on 01-04-2020, University of Oslo, Oslo, Norway) [30].

## 3. Results

### 3.1. Identification of Bacterial Taxa

By plating the sediment samples on contaminated agar, four mixed cultures were isolated and referred to as Consortium 1, Consortium 2, Consortium 3, and Consortium 4 (Table 1).

**Figure 2:**
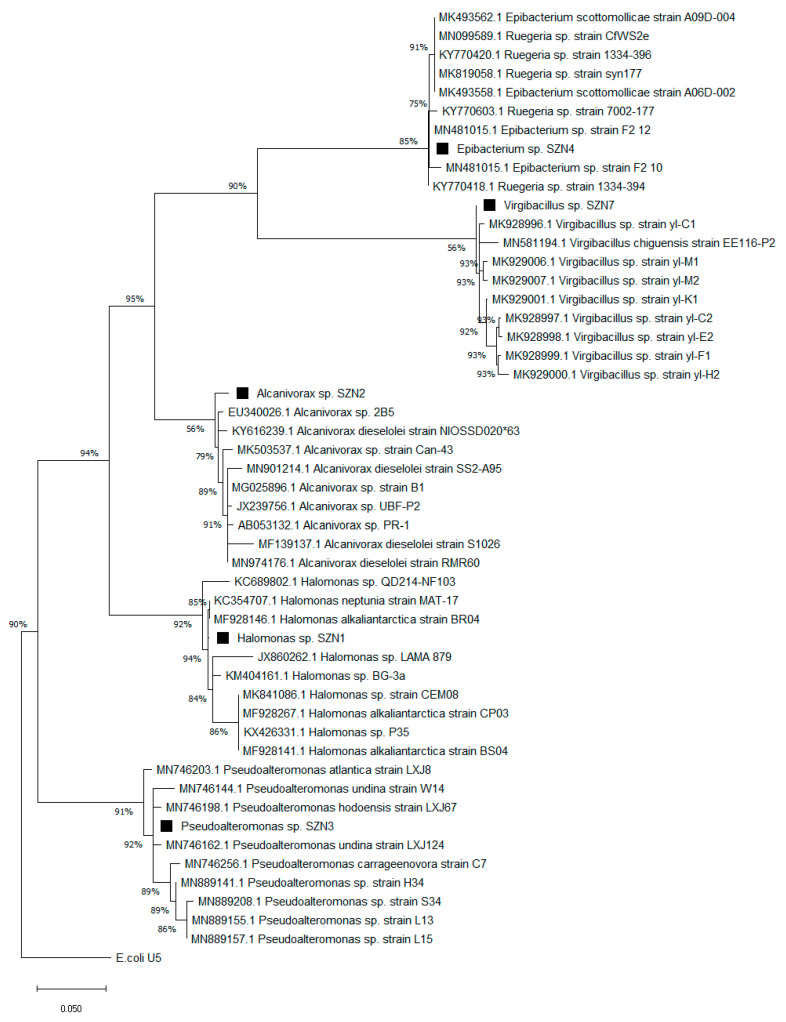
Maximum likelihood tree of the 16S rRNA gene sequences of the five cultured taxa isolated from Bagnoli–Coroglio sediments (*Halomonas* sp. SZN1, *Alcanivorax* sp. SZN2, *Pseudoalteromonas* sp. SZN3, *Epibacterium* sp. SZN4, and *Virgibacillus* sp. SZN7) and their closest cultured representatives with validly published names. For phylogeny test and tree construction, MEGA 7 was used [25]. Scale bar indicates 0.05 substitutions per position.

Consortium 1 was composed of *Halomonas* sp. SZN1 and *Alcanivorax* sp. SZN2 (Figure 2) Consortium 2 was composed of *Pseudoalteromonas* sp. SZN3 and *Alcanivorax* sp. SZN2; Consortium 3 of *Halomonas* sp. SZN1, *Pseudoalteromonas* sp. SZN3, and *Virgibacillus* sp. SZN7; and the Consortium 4 of *Epibacterium* sp. SZN4, and *Halomonas* sp. SZN1.

### 3.2. Hydrocarbon Removal

The concentration of the different hydrocarbons in the sampled sediment used for the experiments is reported in Table 2. A total of nine compounds were detected. From the control experimental conditions, comparing polycyclic aromatic hydrocarbons (PAHs) concentrations at the beginning and end of incubation time, we assessed that PAHs did not interact significantly with plastic of the recipient or with the culture medium with variations in PAHs concentration ranging between −15% and +6%.

**Figure 3:**
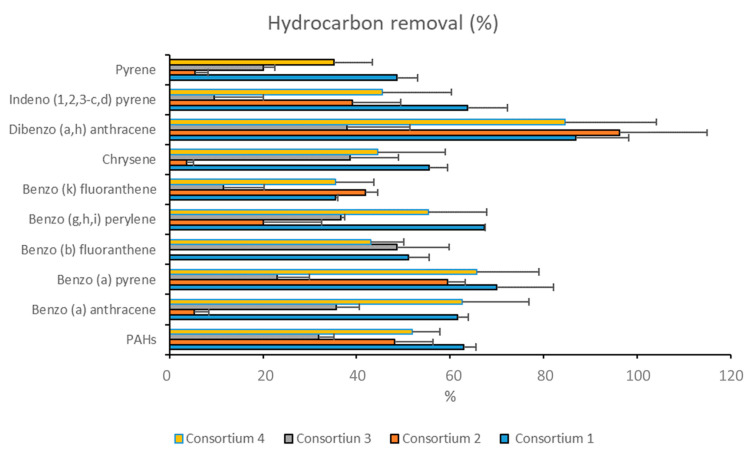
Hydrocarbon removal rates (%) after 27 days of incubation of polluted sediments with the four consortia (see Table 1 for bacterial composition).

All consortia were able to reduce the concentrations of hydrocarbons present in the sediments (Figure 3). The percentage of removal for each PAH congener ranged from 5% (pyrene, chrysene, and benzo(a)anthracene) to 86% (dibenzo anthracene) (Figure 3).

Each consortium showed different degradation rates for the different hydrocarbons (Figure 3). Consortium 1 (*Halomonas* sp. SZN1 and *Alcanivorax* sp. SZN2) displayed the highest hydrocarbon removal efficiency with a mean degradation rate of 63% ± 12.8%. The maximum hydrocarbon removal was observed for dibenzo(a,h)anthracene (86% ± 11.3%) while the lowest was recorded for benzo(k)fluoranthene (35% ± 0.5%).

Consortium 2 (*Pseudoalteromonas* sp. SZN3 and *Alcanivorax* sp. SZN2) showed a degradation capacity of 96% ± 18.7% for dibenzo(a,h)anthracene, 59% ± 3.9% for benzo(a)pyrene, 48% ± 8.2% for PAHs, 41% ± 2.8% for benzo(k)fluoranthene, 39% ± 10.4% indeno (1, 2, 3) pyrene, and 20% ± 12.6% for benzo(g,h,i)perylene, while the degradation of pyrene, chrysene, benzo(b)fluoranthene, and benzo(a)anthracene was almost null (Figure 3).

Consortium 3 (*Halomonas* sp. SZN1, *Pseudoalteromonas* sp. SZN3, and *Virgibacillus* sp. SZN7) was the least effective in removing hydrocarbons since the mean hydrocarbon removal rate was 29% ± 12.8%. The highest degradation rate (48% ± 11.1%) was achieved for benzo(b)fluoranthene, while a lower ability to remove hydrocarbons from sediments was observed for indeno pyrene (9% ± 10.5%), benzo(k)fluoranthene (11% ± 8.7%), and pyrene (20% ± 2.5%) (Figure 3).

Consortium 4 (*Epibacterium* sp. SZN4 and *Halomonas* sp. *SZN1*) showed an overall degradation rate of 50% ± 17.6%. Higher removal rates were recorded for dibenzo anthracene (84% ± 19.6%), benzo anthracene (62% ± 14.2%), and benzo(a)pyrene (65% ± 13.3%). Conversely, lower degradation rates were observed for pyrene (20% ± 8.2%) and benzo(k)fluoranthene (35% ± 8.3%) (Figure 3).

**Figure 4:**
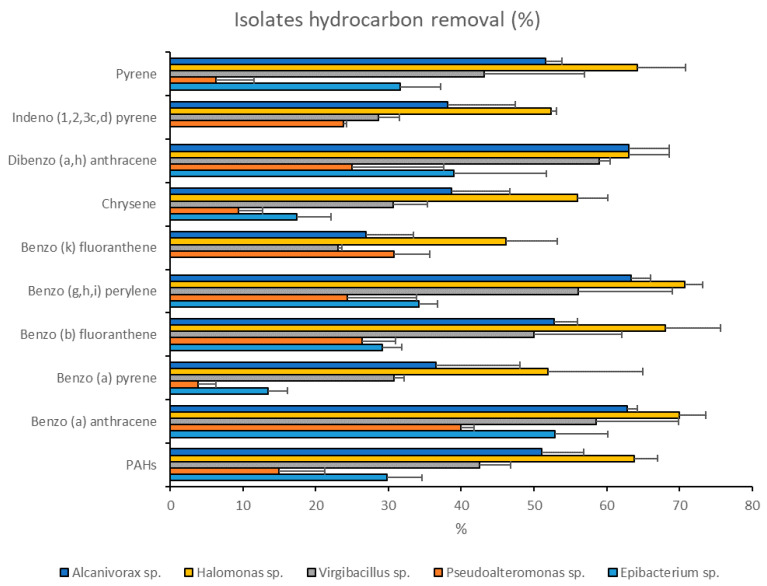
Hydrocarbon removal (%) after 27 days of incubation of polluted sediments with the five single taxa, *Halomonas* sp. SZN1, *Alcanivorax* sp. SZN2, *Pseudoalteromonas* sp. SZN3, *Epibacterium* sp. SZN4, and *Virgibacillus* sp. SZN7.

The two strains, *Halomonas* sp. SZN1 (belonging to consortia 1, 3, and 4) and *Alcanivorax* sp. SZN2 (belonging to consortia 1 and 2) had the highest degradation rates, with an average removal of 60% ± 8.5% and 48% ± 12.9%, respectively (Figure 4).

*Halomonas* sp. SZN1 achieved high hydrocarbon removal for benzo(g,h,i)perylene and benzo anthracene (70% ± 2.5% and 70% ± 3.7%, respectively), while the lowest removal rate was observed for benzo(k)fluoranthene (46% ± 7.1%).

*Alcanivorax* sp. SZN2 exhibited relatively high hydrocarbon removal capacity (63% ca.) for dibenzo anthracene (63% ± 5.6%), benzo(g,h,i)perylene (63% ± 2.6%) and benzo anthracene (62% ± 1.4%), whereas it showed a low ability towards benzo(k)fluoranthene (26% ± 6.5%).

By contrast, the single bacterial isolate *Pseudoalteromonas* sp. SZN3 (present in consortia 2 and 3) was the least effective in the degradation of hydrocarbons, exhibiting a mean removal efficiency of 20% ± 11.7%. While the removal rate of benzo anthracene reached 40% ± 1.8%, very low removal rates (<10%) were reported for benzo(a)pyrene (3% ± 2.4%) and pyrene (6% ± 5.2%) (Figure 4).

*Epibacterium* sp. SZN4 showed an effective removal, between 30% and 50% for the majority of the analysed compounds, with a mean removal rate of 31% ± 12.2%. The highest removal efficiency was found for benzo anthracene (52% ± 7.3%) while benzo(a)pyrene and chrysene degradation rates did not exceed 20% (Figure 4). *Epibacterium* sp. SZN4 did not affect indeno pyrene and benzo(k)fluoranthene concentrations.

*Virgibacillus* sp. SZN7 showed an overall capability of reducing hydrocarbon concentrations to 42% ± 13.6% and benzo(k)fluoranthene, the least-degraded compound, to 23% ± 0.5% (Figure 4). However, this species induced removal rates of 59% ± 1.4% and 58% ± 11.4%, respectively, in the case of indeno pyrene and benzo anthracene.

### 3.3. Heavy Metal Immobilization

**Figure 5:**
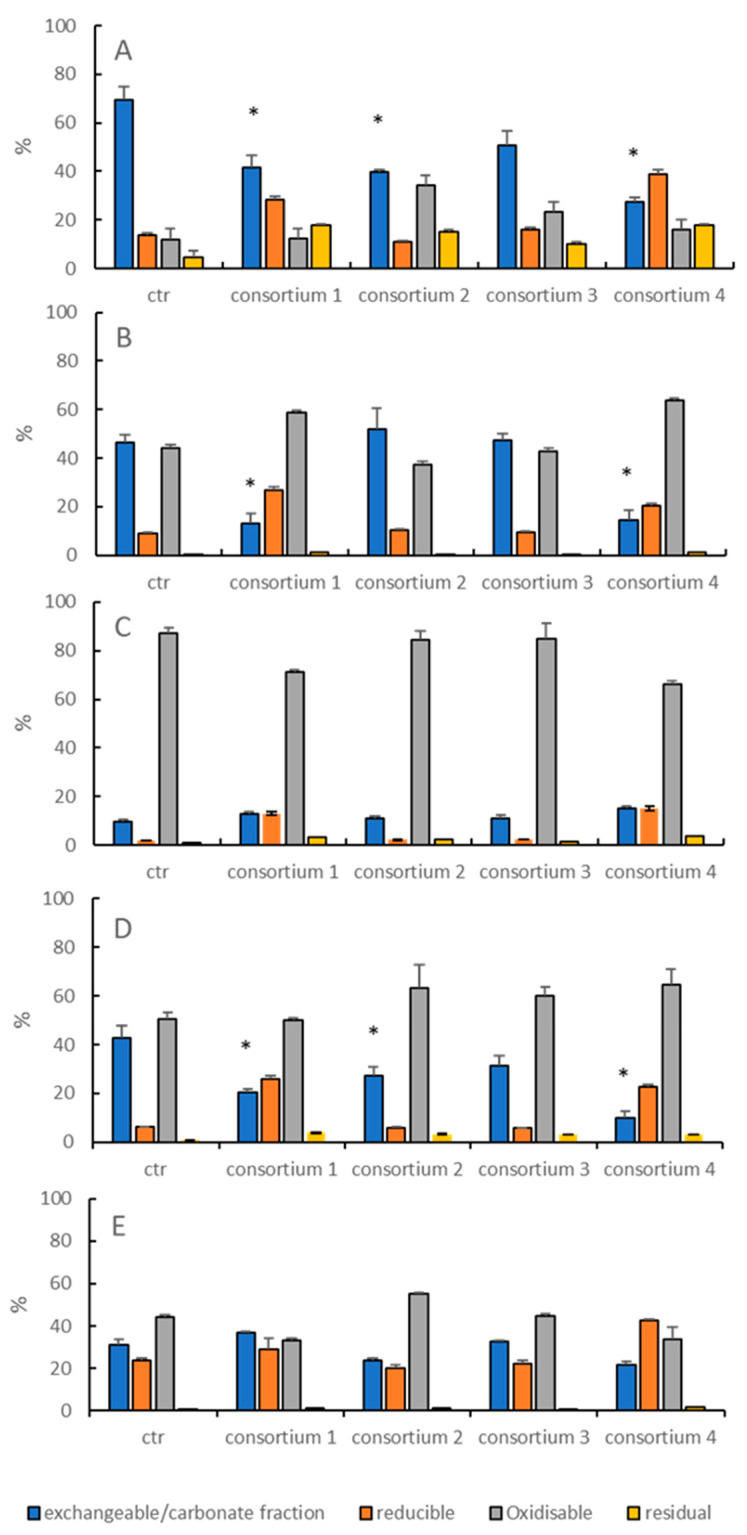
Heavy metal distribution in the four fractions (exchangeable/carbonate, oxidisable, reducible, and residual fractions) after 27 days of incubation of polluted sediments in the presence of the four mixed cultures (see Table 1 for bacterial composition) and in the control microcosm (ctr). Panel (**A**), arsenic; (**B**), cadmium; (**C**), copper; (**D**), lead; (**E**), zinc. The asterisk (*) indicates significant difference between treatments and control.

With regards to heavy metal immobilization, Consortium 1 was effective in reducing the amount of metals associated with the carbonate/exchangeable fraction, with a reduction of 40%, 73% and 53% for As, Pb, and Cd, respectively (Figure 5). Consortium 2 was effective on As and Cd geochemical partitioning by reducing the amount of metals associated with the most bioavailable fractions by 44% and 36%, respectively. Consortium 4 was the most effective in reducing As and Cd bioavailability reaching values of 61% and 76%, respectively. Moreover, 71% of the Pb bound to the carbonate/exchangeable fraction was reduced. Conversely, Consortium 3 did not display any effect on metal partitioning among the different geochemical fractions.

**Figure 6:**
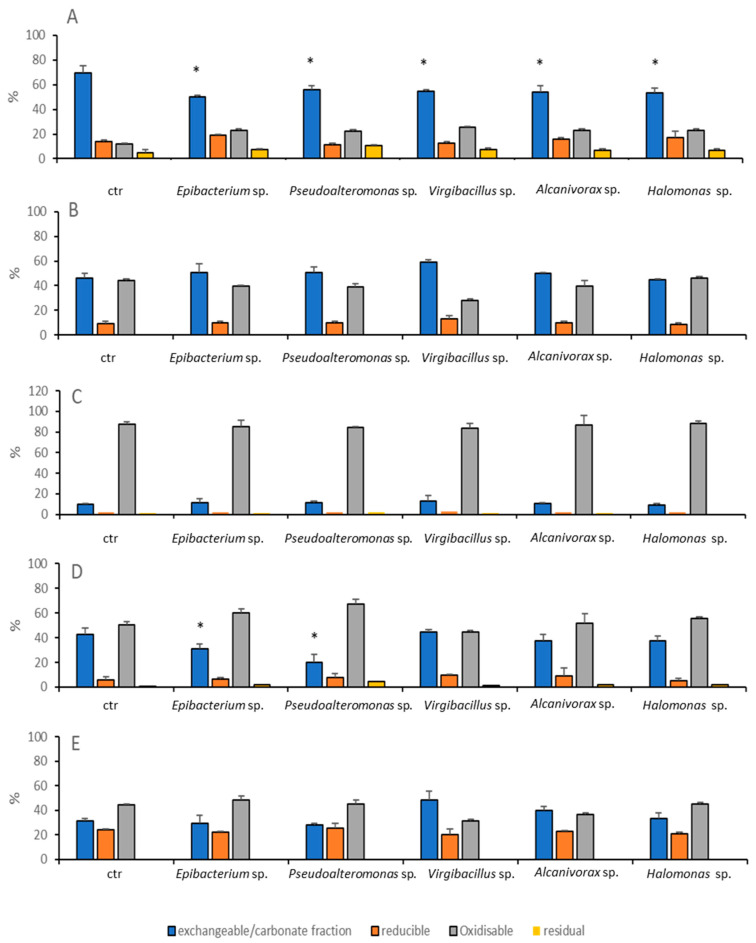
Heavy metal distribution in the four fractions (exchangeable/carbonate, oxidisable, reducible, and residual fractions) after 27 days of incubation of polluted sediments with the five bacterial strains: *Epibacterium* sp. SZN4, *Pseudoalteromonas* sp. SZN3, *Virgibacillus* sp. SZN7, *Alcanivorax* sp. SZN2, and *Halomonas* sp. SZN1 and in the control (ctr). Panel (**A**), total arsenic; (**B**), cadmium; (**C**), copper; (**D**), lead; (**E**), zinc. The asterisk (*) indicates significant difference between treatments and control.

As pure cultures, the isolated strains only affected As and Pb partitioning (Figure 6). By contrast, Cu and Zn were not affected by any of the bacteria investigated.

*Epibacterium* sp. SZN4, *Pseudoalteromonas* sp. SZN3, *Virgibacillus* sp. SZN 7, *Alcanivorax* sp. SZN2 and *Halomonas* sp. SZN1 lowered the concentration of As bound to the carbonate/exchangeable fraction by 28%, 20%, 20%, 22%, and 23%, respectively. Pb was only affected by treatments with *Epibacterium* sp. SZN4 and *Pseudoalteromonas* sp. SZN3 that decreased its bioavailability by 24% and 52%, respectively.

## 4. Discussion

Table 2 allowed the taxonomic identification of five bacteria isolated from polluted sediments. Interestingly, although both *Halomonas* sp. SZN1 and *Alcanivorax* sp. SZN2 belong to genera already described as capable of degrading hydrocarbons [31,32], they do not cluster closely with species with hydrocarbon degrading activity. Similarly, *Pseudoalteromonas* sp. SZN3 differs greatly in terms of homology from its closest phylogenetic neighbours, *Pseudoalteromonas nigrifaciens* and *P. elyakovii*, whose 16s sequences have been found associated with bacterial consortia having hydrocarbon degrading activity [33]. Furthermore, although members belonging to the genus *Virgibacillus* have already been described as involved in the metabolism of hydrocarbons [34], *Virgibacillus* sp. SZN7 showed a close homology with sequences belonging to *Virgibacillus pantothenticus* and *V. byunsanensis*, both of which are not associated with the degradation of hydrocarbon compounds. Finally, to our knowledge, the identification of a member belonging to *Epibacterium* genus (*Epibacterium* sp. SZN4) involved in hydrocarbon removal mechanisms had not yet been described in the literature.

The mean hydrocarbon concentrations removed for the mixed cultures tested in this study is in agreement with the degradation rate of about 50% reported previously for a consortium composed of *Pseudomonas aeruginosa*, *Marinobacter mobilis*, *Gaetbulibacter* sp. and *Halomonas* sp. [35]. The effectiveness of a consortia composed mainly by *Halomonas* sp. SZN1, *Alcanivorax* sp. SZN2 and *Pseudoalteromonas* sp. SZN3, in degrading both aliphatic hydrocarbons and PAH in this study is in agreement with previous studies [36] in which a consortium consisting of such taxa was able to degrade up to 40% of the contaminating oil added to mesocosms.

The concomitant presence of *Halomonas* sp. and *Alcanivorax* sp. in Consortium 1 in sediments contaminated with hydrocarbons has been reported before [37], due to the known ability of representatives of these genera to metabolise hydrocarbons [38,39]. The two strains in our study were able to degrade almost all hydrocarbons with a high efficiency when co-cultured, especially indeno(1,2,3,)pyrene, dibenzo(a)anthracene, and benzo(a)pyrene. These results reveal the high potential synergistic degradation rate of these two strains when co-cultured.

The isolation of a *Pseudoalteromonas* strain SZN3 from polluted sediments is consistent with data reported in the literature [40,41] reporting that *Pseudoalteromonas* spp. plays a predominant role in the degradation of hydrocarbons and in the reduction of metal toxicity, for example through the presence of mercury-resistant operons whose presence has been described in *Pseudoalteromonas haloplanktis* [42]. However, in our study *Pseudoalteromonas* strain SZN3 demonstrated lowered degradation capacity, which resulted in the decreased efficiency of Consortium 2 (*Pseudoalteromonas* sp. SZN3 and *Alcanivorax* sp. SZN2) in degrading hydrocarbons compared to Consortium 1. Despite that *Pseudoalteromonas* sp. has a predominant role in hydrocarbon and heavy metal remediation, it is known to efficiently degrade hydrocarbons in association with other bacteria [33,43,44], and from our study co-culturing with *Alcanivorax* sp. SZN2 does not appear to result in the most efficient complementation. This result highlights the relevant role of cooperation of bacteria in consortia to degrade hydrocarbons. It is possible that mutualistic interactions can occur between different components of microbial communities, since the exometabolites produced by a particular taxon, not necessarily directly involved in the metabolism of pollutants, can be used by other organisms with high degrading capacities to increase their metabolic capacities [45].

Few studies report the presence of *Epibacterium* sp. in contaminated sediments probably because many of these strains are usually identified as belonging to the clade of Ruegeria. Both genera belong to the Roseobacter lineage, which have been suggested to be a possible hydrocarbon degrader as revealed by the presence of many genes in their genome encoding for alkane hydroxylases and uncharacterised ring-cleaving and ring-hydroxylating dioxygenases [46]. Indeed, previous studies [18,47,48] highlighted how Roseobacter and specifically *Ruegeria* sp. are able to favour the degradation of hydrocarbons. The hydrocarbon removal capacity of *Epibacterium* sp. SZN4 confirms the effectiveness of Rhodobacteraceae members, such as *Ruegeria* sp., present in a wide range of marine habitats [49], to deal with such organic pollutants [50].

*Virgibacillus* sp. has been associated with polluted sediments in a few studies, [51], while the taxa belonging to Virgibacillus possess enzymes involved in catechol degradation [34]. These results encourage further investigations on the ability of this taxon to degrade hydrocarbons since the sequences belonging to the genus Virgibacillus have been shown to be dominant in bacterial communities associated with petroleum contaminated desert soil [52]. Furthermore, its ability to produce bio-flocculating compounds to enhance hydrocarbon biodegradation and metal ion removal [53,54] represents another key factor for this promising bacterium for bioremediation purposes.

The ability of microorganisms to reduce concentrations of soluble/exchangeable metals is of significant interest, as it is known that metals that bind with organic matter or form inorganic precipitates (e.g., sulphides) have a lower mobility and decreased toxicity [55]. This ability to immobilise metals by precipitating them to insoluble forms in the sediment has been described for sulphate-reducing bacteria, which are able to reduce the amount of metals associated with the exchangeable fraction by up to 70% [56,57,58,59].

Sulphate-reducing bacteria are not the only bacteria able to induce changes in the partitioning of metals into less mobile geochemical fractions [60], through biosorption and bioaccumulation [61,62], or in acid environments, through bioleaching [63]. The present study confirms the ability of bacteria mainly belonging to the order Oceanospirillales, Rhodobacterales, and Alteromonadales, to effectively lower metal bioavailability. Moreover, experimental data reveal that the highest reduction in metal mobility is obtained using co-cultures rather than individual isolates.

Arsenic mobility was reduced by all bacterial strains as well as by the consortia in the present study. This capacity is probably due to the large fraction of As associated with the most mobile fraction as well as its widespread occurrence throughout the sampled area [64]. *Halomonas* sp. showed a poor capacity to reduce As bioavailability, which increased when *Halomonas* sp. SZN1 and *Alcanivorax* sp. SZN2 were associated to this species, enhancing their biological activity towards metals. Conversely, the distribution of other metals, such as Cu and Zn was not modified by any of the bacteria strains, since the mobile fraction of such elements was reduced compared to reducible and oxidisable fractions.

*Epibacterium* sp. SZN4 and *Pseudoalteromonas* sp. SZN3 were the only isolated strains able to reduce the mobility of Pb. The ability of the *Pseudoalteromonas* sp. SZN3 is not surprising since it has been demonstrated that the production of glycoprotein exopolymers by *Pseudoalteromonas* sp. induces metal ion binding [65]. While previous studies [66] report a decreased Pb mobility promoted by the activity of Oceanospirillaceae, Sinobacteraceae, Flavobacteriaceae, Firmicutes, and Bacteroidetes, to our knowledge, this is the first time that members of Epibacterium and Pseudoalteromonas genera are described to efficiently partition Pb when co-cultured.

Except for As, *Virgibacillus* sp. SZN7 (belonging to Consortium 3) does not affect the distribution of any of the other metals. Such an observation may explain the reduced ability of Consortium 3 to change metal partitioning among the different geochemical fractions of the sediment.

Our data indicate that bacteria that do not belong to the Deltaproteobacteria class, widely recognised as comprising most of the sulphate-reducing bacteria [67], are able to lower the toxicity of metals by increasing their immobilization. In general, reduced metal mobility following bacterial consortia addition is comparable to the activity of the already described sulphate reducing bacteria [68], which were reported to reduce mobility for Cu, Cd, Zn and Pb in a range between 20% and 60%.

Finally, our results highlight that mutualistic interactions within mixed cultures demonstrate synergistic capacity in reducing metal mobility (except Consortium 3), which out-performs the reduction induced by the individual isolates.

We are aware that the transposition of the results here documented from an ex-situ study (amending natural contaminated sediments with their bacteria hosts with enriched culture medium) to natural environments is not fully realistic [69]. However, our study, in comparing the performance of different natural consortia and single strains to deal with natural contaminated sediments, paves the way to select some of them for further in situ simulated studies.

## 5. Conclusions

Our results denote the capability of five bacteria, individually and as mixed cultures, to degrade PAHs and reduce the mobility of arsenic, lead, and cadmium. PAHs degradation rates depend on the type of hydrocarbons, bacterial strain and their presence as a co-culture or as a pure culture. Among the five strains analysed, *Halomonas* sp. SZN1 was the most efficient, either as a pure inoculum, or in co-culture with *Alcanivorax* sp. Benzo anthracene, benzo(g,h,i)perylene, and benzo(b)fluoranthene were the hydrocarbons most efficiently degraded, suggesting the potential of these organisms (*Halomonas* sp. SZN1 and *Alcanivorax* sp. SZN2) to be developed as an effective treatment of polluted sediments ex situ and in situ. Results of selective sequential extractions (SSE) concerning metal partitioning in geochemical fractions suggest that Consortia 1, 2, and 4 may represent promising tools to decrease As, Pb, and Cd toxicity, with *Pseudoalteromonas* sp. SZN3 and *Epibacterium* sp. SNZ4 are the most efficient in lowering the mobility of As and Pb.

This study highlights the potential use of bacterial strains, especially *Halomonas* sp. SZN1 and *Alcanivorax* sp. SZN2 and three Consortia (1, 2 and 4) isolated from chronically contaminated sediments for improving the effectiveness of bioremediation strategies towards hydrocarbons and heavy metals. Moreover, we show that single isolates (principally *Halomonas* sp.) might perform equally or even better than the related consortia. We also demonstrate that some bacteria, e.g., *Pseudoalteromonas* sp., *Epibacterium* sp., or *Virgibacillus* sp., and their related consortia, possess the ability to remove hydrocarbons and immobilise metals. Finally, we show that *Alcanivorax* sp. SZN2 strain was able to degrade PAHs, even though members of the *Alcanivorax* group are principally known to degrade alkanes. This study paves the way for further investigations still required to implement bioremediation interventions using those strains. Metabolomics and genomics integrated study on those strains, both single and consortia, will give insights on the regulatory mechanisms explaining PAHs degradation and metals bioavailability reduction observed in our study. Furthermore, the proteome study of the single isolates used in this study will allow to identify and characterise possible new enzymes of interest for bioremediation treatments. All this information is a requisite for further developing biotechnological challenges of these bacteria.

## Figures and Tables

**Table 1:** Bacterial composition of the four consortia.

Consortia	Culturable Strains
Consortium 1	*Halomonas* sp. SZN1 *Alcanivorax* sp. SZN2
Consortium 2	*Pseudoalteromonas* sp. SZN3 *Alcanivorax* sp. SZN2
Consortium 3	*Halomonas* sp. SZN1 *Pseudoalteromonas* sp. SZN3 *Virgibacillus* sp. SZN7
Consortium 4	*Epibacterium* sp. SZN4 *Halomonas* sp. SZN1

**Table 2:** Hydrocarbon concentrations (mg kg^−1^) in the sediment before the experiments. Reported values are mean and standard deviations (SD). PAH = polycyclic aromatic hydrocarbon.

PAH Congeners	Mean	SD
Benzo anthracene	62.5	11.7
Benzo(a)pyrene	105.3	19.4
Benzo(b)fluoranthene	54.3	5.1
Benzo(g,h,i)perylene	57.5	10.4
Benzo(k)fluoranthene	75.8	7.6
Chrysene	132.5	12.6
Dibenzo anthracene	11.9	16.0
Indeno pyrene	48.8	13.8
Pyrene	210	21.6
